# Detection of feline morbillivirus in cats with symptoms of acute febrile infection

**DOI:** 10.1007/s11259-023-10214-x

**Published:** 2023-09-06

**Authors:** Genta Ito, Shoichi Tabata, Aya Matsuu, Hitoshi Hatai, Yuko Goto-Koshino, Tomohide Kuramoto, Sakiko Doi, Yasuyuki Momoi

**Affiliations:** 1 Department of Veterinary Clinical Pathobiology, Graduate School of Agricultural and Life Sciences, The University of Tokyo, 1-1-1 Yayoi, Bunkyo-ku, Tokyo, 113-8657 Japan; 2 Laboratory of Veterinary Diagnostic Imaging, Joint Faculty of Veterinary Medicine, Kagoshima University, 1-21-24 Korimoto, Kagoshima, 890-0065 Japan; 3 Transboundary Animal Diseases Research Center, Joint Faculty of Veterinary Medicine, Kagoshima University, 1-21-24 Korimoto, Kagoshima, 890-0065 Japan; 4 Laboratory of Veterinary Histopathology, Joint Faculty of Veterinary Medicine, Kagoshima University, 1-21-24 Korimoto, Kagoshima, 890-0065 Japan; 5 Present Address: Farm Animal Clinical Skills and Disease Control Center, Faculty of Agriculture, Iwate University, 3-18-8 Ueda, Morioka, Iwate, 020-8550 Japan; 6 Veterinary Medical Center, Graduate School of Agricultural and Life Sciences, The University of Tokyo, 1-1-1 Yayoi, Bunkyo-ku, Tokyo, 113-8657 Japan; 7 Kagoshima University Veterinary Teaching Hospital, 1-21-24 Korimoto, Kagoshima, 890-0065 Japan; 8 Sanritsu Zelkova Co., Ltd., 3-5-5 Ogibashi, Koto-ku, Tokyo, 113-0011 Japan

**Keywords:** Acute infection, Feline morbillivirus, Fever, Leukopenia, Thrombocytopenia

## Abstract

Feline morbillivirus (FeMV) was identified for the first time in cats in 2012 in Hong Kong. Although its association with chronic kidney disease in cats has attracted the attention of researchers, its clinical significance as an acute infection has not been reported. Previously, we reported FeMV detection using next-generation sequence-based comprehensive genomic analysis of plasma samples from cats with suspected acute febrile infections. Here, we conducted an epidemiological survey to detect FeMV by quantitative reverse transcription polymerase chain reaction (qRT-PCR) using blood samples from cats in Japan. FeMV was detected in 32/102 blood samples (31.4%) from cats with suspected acute viral infections. Most of the FeMV-positive cats had clinical findings consistent with acute viral infections, including fever, leukopenia, thrombocytopenia and jaundice. No FeMV was detected in healthy cats or clinically ill cats that visited veterinary hospitals. Phylogenetic analysis classified FeMV L genes into various FeMV subtypes. We also necropsied a FeMV-positive cat that died of a suspected acute infection. On necropsy, FeMV was detected in systemic organs, including the kidneys, lymph nodes and spleen by qRT-PCR and immunohistochemical staining. These results suggest that FeMV infections may cause acute symptomatic febrile infections in cats. A limitation of this study was that the involvement of other pathogens that cause febrile illnesses could not be ruled out and this prevented a definitive conclusion that FeMV causes febrile disease in infected cats. Further studies that include experimental infections are warranted to determine the pathogenicity of FeMV in cats.

Feline morbillivirus (FeMV) was first isolated from stray cats in Hong Kong in 2012 (Woo et al. 2012). Since its discovery, FeMV has been detected in cats in many countries and regions worldwide (Choi et al. 2020; Darold et al. 2017; De Luca et al. 2020; Mohd Isa et al. 2019; Sharp et al. 2016; Yilmaz et al. 2017). FeMV is classified into the genus *Morbillivirus.* The genus includes the measles, canine distemper, and rinderpest viruses, which typically cause febrile acute infections in their hosts. FeMV strains are classified into genotypes 1 and 2. Genotype 1 is further divided into three subtypes: FeMV-1A, FeMV-1B and FeMV-1C. Little is known about the symptoms of FeMV infection in cats. FeMV is detected primarily in the urine and renal tissues and may persist in the urinary system of cats (Furuya et al. 2014; Mohd Isa et al. 2019). The association between FeMV infection and chronic kidney disease has attracted the attention of researchers. However, the results of epidemiological studies have been mixed (Busch et al. 2021; De Luca et al. 2020; Donato et al. 2021; McCallum et al. 2018). Unlike other morbilliviruses, acute natural infections have been infrequently reported, with only two cases in cats reported at necropsy (Chaiyasak et al. 2022). FeMV is rarely detected in the blood. In a previous study, we surveyed cats with clinical signs of acute infection to detect unknown viruses by next-generation sequencing and detected FeMV in some cases (Momoi and Matsuu 2021). Since then, we have continued to perform comprehensive analyses and have noted that FeMV is frequently detected in cats with acute febrile disease. In this study, we hypothesized that FeMV causes acute infections and conducted an epidemiological study using clinical samples from cats with suspected acute febrile infections.

We used 102 clinical samples sent for diagnostic testing for severe fever with thrombocytopenia syndrome (SFTS) by veterinary hospitals in Japan from 2018 to 2019. SFTS is an endemic, zoonotic, febrile disease caused by Huaiyangshan banyangvirus in East Asia (Xu et al. 2011; Yu et al. 2011). Cats are susceptible to the SFTS virus, and most infected cats develop severe febrile disease and a high fatality rate of 62.5% has been reported (Matsuu et al. 2019). The samples were sent from various areas in Japan, but mainly western Japan, particularly the Kyushu region, which is an SFTS-endemic area. The clinical veterinarians briefly described clinical symptoms and clinical test values of the cases. Most of the cats from which these samples were obtained had symptoms such as fever, leukopenia, thrombocytopenia, and jaundice, which were consistent with SFTS. Parvovirus-infected cats often present with SFTS-like fever, leukopenia and thrombocytopenia. Parvovirus is often detected in our comprehensive virus analyses of specimens that have been requested for SFTS testing. In parvovirus infections, the virus is detected in the blood. SFTS virus and parvovirus were tested by polymerase chain reaction (PCR), and only specimens that tested negative were used to detect FeMV infection. Feline calicivirus infection and feline infectious peritonitis are often diagnosed as febrile viral infections in cats. However, we did not test for these infections in this study because only blood samples were available. For some samples, FIV/FeLV test results were provided by the veterinarians. As controls, 53 samples stored at -20°C from cats without symptoms of acute infection were kindly provided by a veterinary hospital located in an SFTS-endemic area (Kagoshima Prefecture, Japan). Other control samples were from 99 sick cats visiting Kagoshima University Veterinary Teaching Hospital located in an endemic area, and 222 sick cats visiting Tokyo University Veterinary Teaching Hospital (metropolitan area). These samples were randomly selected from the remaining blood samples collected for clinical examinations from 2020 to 2021.

Nucleic acids were extracted from plasma samples. Real-time PCR was performed using two primers (5′-GGGATCCAGAGGGTAACCT-3′ and 5′-CCGGCCATTAATCTCTGAA-3′) and a probe (FAM-TATTCGAAAGCGATGATGATGAAAACCATTA-TAMRA) targeting the L gene of FeMV with the Thunderbird Probe One-step qRT-PCR Kit (Toyobo, Osaka, Japan). PCR was conducted in duplicate with the following conditions: reverse transcription at 55°C for 10 min, followed by 95°C for 15 s, and 60°C for 45 s for 40 cycles. The cases were considered FeMV-positive if amplification was observed in both test samples in any cycle (up to 40). The average Ct value of two samples was used as the Ct value. FeMV was detected in 32 of the 102 samples from cats with suspected SFTS (Table 1). The Ct values of the FeMV-positive samples ranged from 27.4 to 39.0. None of the samples from any other group were FeMV positive. Among the control specimens, the group provided by a veterinary hospital in an SFTS-endemic area had a similar risk of exposure to field pathogens as the specimens obtained from cats tested for SFTS. The cats in this group were family owned and presented to the veterinary clinic for non-febrile causes; all cats were allowed outdoors. Information on housing status was available for 84 of the cats tested for SFTS, and 68 were allowed outdoors. For the cats whose sex was known, there were 51 males and 35 females in the SFTS-tested group, and 32 males and 20 females (1 unknown) in the control group, which was not a significant difference between the two groups (χ^2^=0.068, *P*=0.74). The average age of the SFTS-tested group was 5.2 years, which was significantly older than 3.5 years in the control group (Mann–Whitney U test, *P*<0.001). The FeMV-positive rate was 31.4% (32/102) in the SFTS-test group and 0% (0/53) in the control group. There was a significant difference in the positive rate between the two groups (χ^2^=21.0, *p*<0.001). These results suggest that FeMV viremia may have been related to the symptoms in the SFTS-tested group.

**Table 1:** Clinical findings for the FeMV-positive cats

Sample ID.	Sex	Age (year)	Clinical symptoms	Body temperature (°C )	White blood cell count (×10^3^/μl) (RI:2.9-17.0)	Platelet count (×10^3^/μl) (RI:151-600)	Ct value of real-time PCR	FeMV subtype	Anti-FeMV Antibody	Breeding Environment	Residential area	Remarkes
2018-20	MN^*3^	13	Anorexia, lethargy, fever, melena, vomit, jaundice	40.0	3.3	83	34.4	1A	0.09 (negative)	Indoor-outdoor	KGS^*9^	FIV(+)
2018-21	FN^*4^	4	Anorexia, lethargy, fever, jaundice	40.0	6.5	157	29.8	1A	0.18 (negative)	Indoor-outdoor	KGS	Oral,rectal swab FeMV(+)
2018-38	MN	3	Anorexia, lethargy, fever, vomit, diahrea	39.3	9.2	94	38.6	1A	0.07 (negative)	Indoor	NGS	FIV(-), FeLV(-)
2018-61	FN	5	Anorexia, lethargy, fever, diahrea	39.9	6.3	113	35.9	ND^*8^	NA	Indoor-outdoor	KGS	
2018-72	MN	7	Anorexia, lethargy, fever, jaundice, rhinorrhea, eye discharge	40.8	17.0	96	38.0	1C	0.09 (negative)	Indoor-outdoor	KGS	
2018-79	MN	6	Anorexia, lethargy, fever, vomit, jaundice	40.4	3.2	13	38.3	1A	NA	Indoor-outdoor	TYM^*10^	FIV(+), FeLV(-),Oral,rectal swab FeMV(+)
2018-107	M^*5^	9 M	Anorexia, lethargy	39.4	3.6	59	37.0	1C	0.35(negative)	Indoor-outdoor	NGS^*11^	FIV(-), FeLV(-)
2018-109	MN	Adult	Anorexia, lethargy, fever, cough, jaundice	41.0	5.7	77	31.1	1A	0.07(negative)	Outdoor	KGS	FIV(+)
2018-111	M	1	Rhinorrhea, eye discharge, rhinorrhea	NA	8.8	105	31.5	1A	0.35(negative)	Outdoor	KGS	FIV(-), FeLV(-), Oral swab FeMV(+)
2018-122	MN	10	Anorexia, lethargy, fever, vomit	40.4	1.9	125	35.3	1A	0.27(negative)	Indoor-outdoor	KGS	
2018-123	N.A.	NA	Anorexia, lethargy, fever	39.1	1.0	129	31.3	ND	NA	Outdoor	KGS	
2018-129	MN	8	Anorexia, lethargy, fever, vomit	40.2	4.5	63	33.0	1A	0.13(negative)	Indoor-outdoor	KGS	
2018-130	F^*6^	1	Anorexia, lethargy, fever, vomit, jaundice	38.3	30.0	0	32.0	1A	0.13 (negative)	Outdoor	FKO^*12^	FIV(-), FeLV(-), Oral swab FeMV(+)
2019-13	F	2	Anorexia, lethargy, fever, jaundice	39.9	18.6	96	35.9	1A	NA	Indoor-outdoor	KGS	FIV(-), FeLV(-), Oral swab FeMV(+)
2019-21	MN	8 M	Anorexia, lethargy, fever, vomit, diarrhea	NA	1.9	NA	38.9	ND	0.23(negative)	Indoor-outdoor	KGS	
2019-27	N.A.	NA	NA	NA	NA	NA	38.1	ND	0.41 (negative)	NA	KGS	
2019-28	F	8	Anorexia, lethargy, fever, vomit, jaundice	39.8	24.3	200	30.0	1A	0.51(negative)	Indoor-outdoor	KGS	
2019-31	MN	13	Anorexia, lethargy, fever, vomit, jaundice, azotemia	36.9	7.8	20	36.9	1A	NA	Indoor-outdoor	KGS	Urine FeMV(-)
2019-36	MN	3	Anorexia, lethargy, fever, vomit, diahrea, salivation	40.0	7.6	121	28.4	ND	0.22 (negative)	Indoor-outdoor	NGS	Oral swab FeMV(+)
2019-37	MN	9	Anorexia, lethargy,vomit, diarrhea	NA	3.3	85	38.9	ND	0.32(negative)	Indoor	KGS	Oral, rectal swab FeMV(-)
2019-41	M	3	Anorexia, lethargy, fever	NA	5.9	90	33.6	1A	0.51(negative)	NA	Saga	
2019-42	FN	5	NA	NA	12.7	134	29.8	1A	0.38(negative)	NA	KGS	SAA 179.2 μg/ml
2019-64	MN	9	Anorexia, lethargy, fever, vomit	39.7	3.4	70	36.2	1A	<0.0 (negative)	Indoor-outdoor	KGS	Seroconverted 5 months later (OD=2.68)
2019-66	FN	1	NA	40.8	NA	NA	36.3	1A	NA	Indoor-outdoor	KGS	
2019-101	FN	12	Anorexia, lethargy, fever	40.5	6.5	159	34.3	1A	0.42(negative)	Indoor-outdoor	KGS	FIV(-), FeLV(-)
2019-108	MN	2	Anorexia, lethargy,diarrhea	38.2	9.1	91	35.2	1A	0.05 (negative)	Indoor-outdoor	KGS	FIV(-), FeLV(-), Seroconverted 2 weeks later (OD=2.03)
2019-115	M	9	Anorexia, lethargy, fever	40.3	6.5	79	30.3	1C	NA	Indoor-outdoor	NGS	FIV(+)
2019-117	FN	7	Anorexia, lethargy, fever, diahrea, vomit, jaundice	40.2	16.3	15	36.9	1A	NA	Indoor-outdoor	KGS	FIV(-), FeLV(-), Oral,rectal swab FeMV(+)
2019-130	F	6	Anorexia, lethargy, fever, vomit, jaundice	39.4	8.7	47	30.4	1B	0.1 (negative)	Indoor-outdoor	NGS	Oral, rectal swab FeMV(+)
2019-138	M	NA	NA	39.4	9.1	148	39.0	1A	NA	Indoor-outdoor	HYO^*13^	
2019-140	FN	10	Anorexia, lethargy, fever, vomit	40.1	13.9	143	32.3	1A	NA	NA	KGS	Oral, rectal swab FeMV(+)
2018-119^*2^	M	1	Anorexia, lethargy, dead on arrival, necropsied	NA	NA	NA	27.4	1A	1.21 (positive)	NA	KGS	FIV(-), FeLV(-), Oral, rectal swab FeMV(+)

^*1^RI:Reference Intervals, ^*2^: Case 2018-119 was necropsied, ^*3^MN: male neuterd, ^*4^MF: female neuterd, ^*5^M: intact male, ^*6^F: intact female, ^*7^NA : data not available, ^*8^ND: not detected, ^*9^KGS: Kagoshima Pref., ^*10^TYM: Toyama Pref., ^*11^NGS: Nagasaki Pref., ^*12^FKO: Fukuoka Pref., ^*13^HYO: Hyogo Pref

The FeMV-positive samples were tested by nested reverse transcription (RT)-PCR targeting the L gene of FeMV, as previously described (Furuya et al. 2014) with some modifications. Two oligonucleotide primers (5’-GGAACATGGCCTCCTGTAGA-3’ and 5’-CTCCATTGGCAATCAGGTTT-3’) were used for reverse transcription at 55°C for 30 min. The first PCR was performed at 95°C for 2 min of heat inactivation, followed by 95°C for 30 s, 55°C for 30 s, and 68°C for 30 s for 40 cycles. The second PCR was performed at 95°C for 2 min of heat inactivation and 95°C for 30 s, 50°C for 30 s, and 72°C for 30 s for 40 cycles using TaKaRa Ex Taq (Takara, Shiga, Japan) with primers 5’-CCAAATCATGCATCTGCTGT-3’ and 5’-GCGAACAATCGACCTACCTC-3’ to amplify the 401-bp DNA fragment. In 27 of the 32 FeMV-positive samples, the FeMV gene was amplified by nested PCR. The PCR products were submitted for direct nucleotide sequencing (Fasmac, Kanagawa, Japan). To evaluate the evolutionary relationships, nucleotide sequences of the detected FeMV gene and FeMV genomes registered in the GenBank database were analyzed using the maximum likelihood tree method to create a phylogenetic tree using MEGA 11 (https://www.megasoftware.net) applying the Tamura 3-parameter model (Tamura 1992) as the best fit nucleotide substitution models using the selection tool in MEGA 11 (Fig. 1). Twenty-two of 27 nested PCR-positive samples, 22 samples were classified into subtype FeMV-1A. One sample was FeMV-1B and three samples were FeMV-1C. Nucleotide sequence data were not obtained for one sample (ID: 2019-21) because of inadequate volume. We also assessed anti-FeMV antibodies using enzyme-linked immunosorbent assay (ELISA) in measurable cases. An artificial gene encoding the nucleocapsid protein (NP) of FeMV was synthesized. The gene was cloned into the bacterial expression plasmid pGEX6p-1 for expression as a fusion protein with glutathione S-transferase (GST). After expressing the GST–NP fusion protein in the BL-21 strain of *Escherichia coli*, the fusion protein was purified using glutathione Sepharose B and used as an antigen. Western blotting was performed using plasma samples from 14 cats, and samples in which a band was detected at a position corresponding to the molecular weight of GST–NP were defined as positive for anti-FeMV antibody. We used purified GST–NP as an antigen to develop an ELISA assay to detect anti-FeMV antibodies. The OD value was measured by ELISA using 100-fold dilutions of plasma samples. The OD value obtained with purified GST as the antigen was subtracted as a non-specific reaction. We used the western blotting results described above as a gold standard, and when the subtracted value exceeded 0.611, it was considered antibody positive. Antibodies were evaluated in 22 of the 32 PCR-positive cases. Of these, only one (ID: 2018-119), from an necropsied cat was positive (Table 1). Paired sera were available for two of the negative cases. Blood samples were taken from one case 5 months after the PCR test (2019-64), and 2 weeks after the test in the other case (2019-108). The antibody titers were positively converted in each case. Antibodies were evaluated in 68 of 70 PCR-negative cases, and seven10.3% were antibody positive.

**Fig. 1:**
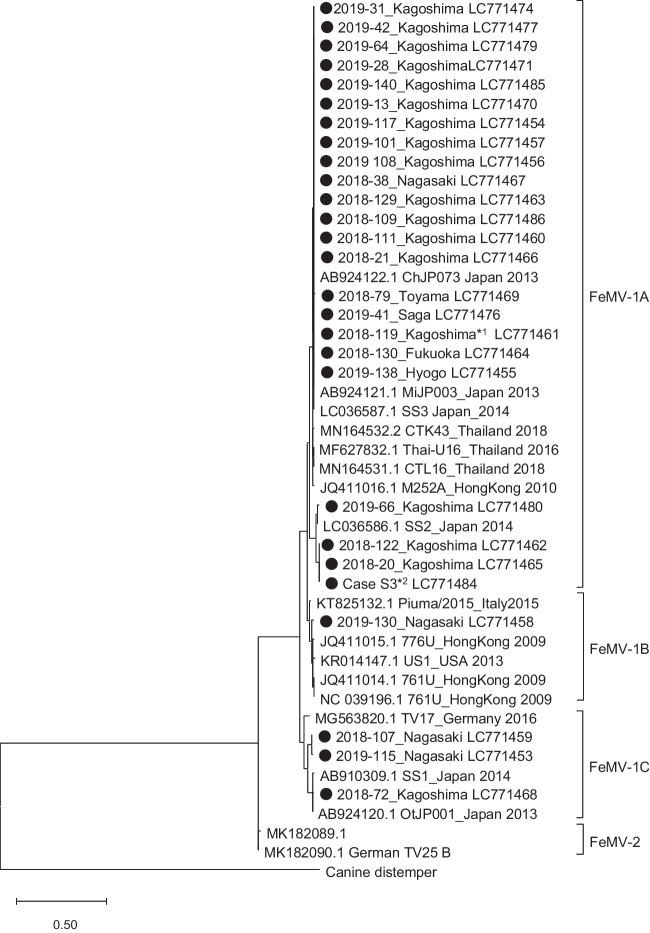
Phylogenetic analysis of the feline morbillivirus partial L segment. Neighbour-joining trees of nucleotide sequences of the partial L segment were constructed using MEGA software. The sequences obtained in this study are shown in black circles. The number written after the sample name is the accession number of DNA Data Bank of Japan (DDBJ). *1: Case #2018-119 was necropsied. *2: Case S3: sequences obtained by next-generation sequencing

Table 1 summarizes the clinical findings of the 32 FeMV-positive cats. Seventeen (53.1%) had a fever of ≥39.5°C. Although the white blood cell counts varied, seven of these 32 cats (21.9%) had marked leukopenia of ≤5000/μL. Sixteen of the 32 cats (50%) had thrombocytopenia of ≤100,000/μL. Twenty-six cats (81.3%) were reported to be allowed to go outdoors.

A necropsy was performed on a 1-year-old male FeMV-positive cat (ID: 2018-119). The cat had begun to show lethargy and anorexia 4 or 5 days previously and died while being transported to a veterinary hospital. Examination for infectious diseases and necropsy were conducted with the owner’s consent. RNA was extracted from the organs and real-time RT-PCR was performed to detect the FeMV gene using the above-mentioned method. The FeMV gene was detected in various tissues with low Ct values, including rectal swab (Ct=24.5), oral swab (29.0), liver (23.3), spleen (17.7), lung (22.1), blood (24.4), kidney (25.3), urine (23.6), and lymph nodes (34.5), indicating severe viremia. Collected tissues were fixed in 10% buffered formalin, routinely processed, and embedded in paraffin wax. For polymer-labeled immunohistochemistry, 3-μm-thick tissue sections were deparaffinized and subjected to antigen retrieval by autoclaving (121°C, 15 min) in citrate buffer (pH 6.0). The sections were immersed in 3% hydrogen peroxide in methanol for 10 min and incubated with 10% normal goat serum for 10 min. The sections were incubated with rabbit anti-feline morbillivirus PV-N protein antiserum (1:1000) (Park et al. 2016) at 37°C for 30 min. After processing with a secondary reagent, the reactions were visualized with Simple Stain DAB solution (Nichirei Biosciences, Tokyo, Japan), and counterstained with Mayer’s hematoxylin. Immunostaining of tissue sections showed that the renal distal tubular epithelial cells were positive for FeMV PV-N protein (Fig. 2a). Macrophages and lymphocytes that had infiltrated an inflammatory focus surrounding the immunopositive tubules and were scattered in the renal interstitium showed immunopositivity (Fig. 2b). Lymphoid follicles of the mandibular lymph node, and macrophages and lymphocytes in the splenic lymphoid follicles and subcapsular to the perinodal sinus also showed immunoreactivity (Fig. 2c, d). Gross findings showed pulmonary edema without cardiac abnormalities, including cardiomyopathy, which is common in cats. No other anomalies were found that may have contributed to the death of this cat.

**Fig. 2:**
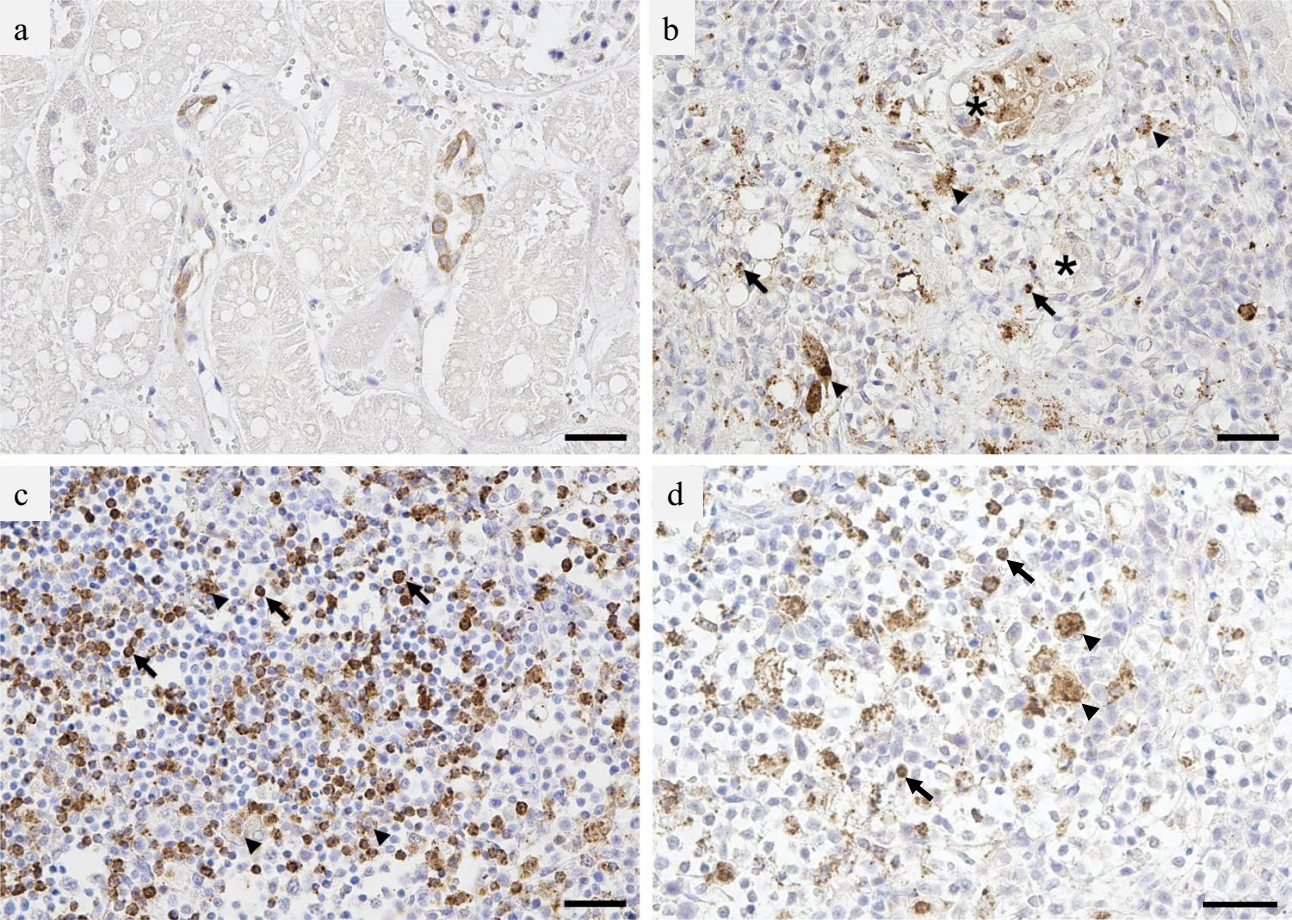
Immunohistochemistry using anti-FeMV PV-N protein antiserum. **a** Kidney showing immunopositivity in the distal tubules. **b** Kidney with infiltrating macrophages (arrow heads) and lymphocytes (arrows) showing immunoreactivity. * distal tubules. **c** Mandibular lymph node, macrophages (arrow heads), and lymphocytes (arrows) showing immunoreactivity. **d** Spleen, macrophages (arrow heads), and lymphocytes (arrows) showing positive for FeMV PV-N. Bar = 30 μm. FeMV, feline morbillivirus

Little is known about FeMV pathogenicity; however, an association with the development of kidney disease has been suggested (Crisi et al. 2020; Sieg et al. 2015; Woo et al. 2012). In the present study, we detected FeMV in plasma samples from cats with symptoms of acute infection. Many of these cats were allowed to go outdoors and were at high risk of exposure to field pathogens. In previous studies, FeMV was primarily detected in urine or kidney samples from asymptomatic cats, and was rarely detected in blood (Mohd Isa et al. 2019; Yilmaz et al. 2017). From our results, we speculated that FeMV can cause viremia with clinical symptoms of acute phase infection and persists in the urinary system. Viral genes were detected with low Ct values in systemic organs (indicating high viral loads) from a dead cat that had symptoms of acute infection. In this young cat, viral antigens were also detected by immunostaining in various tissues. Necropsy revealed no other cause of death; thus, it was likely caused by FeMV infection. To our knowledge, there are no report of cats dying from FeMV infection. In previous epidemiological surveys, FeMV was detected only in asymptomatic cats (Darold et al. 2017; Sharp et al. 2016). Additionally, FeMV was not strongly pathogenic in a previous experimental infection (Nikolin et al*.* 2022). We initially suspected that highly pathogenic viral strains might be emerging in Japan because there are few reports of acute febrile conditions related to FeMV infection. Phylogenetic analysis of the L gene suggested that the detected FeMVs were grouped into various subtypes, and no particular strain was prevalent. This indicated that FeMV detected in this study was not an emerging strain, and that FeMV can cause acute infections in cats. Most of the FeMV-positive samples were from a specific region (Kyushu, SFTS endemic area), which could result in selection bias because of the use of SFTS test specimens.

There is also no information on the clinical symptoms and findings of acute FeMV infection. Many of the FeMV-positive cats in our study had a high fever and jaundice. However, care must be taken in interpreting the results because the findings are common in cats with SFTS and may represent bias resulting from the use of SFTS test specimens. Recently, FeMV was identified in 14 dogs with severe pulmonary disease in Thailand (Piewbang et al. 2022). Another study reported that cats experimentally infected with recombinant FeMV had exhibited symptoms of acute infection, including fever and lymphopenia (Nambulli et al*.* 2022). In the latter study, viral antigens were found mainly in lymphoid tissue, macrophages were the predominant infected cells during acute infection, and FeMV was detected in urine at 12–14 days post-inoculation. The necropsy findings in our study seemed to be consistent with the results of this experimental infection. Viral antigens were also detected in the lungs in the experimental infection study of Nambulli et al. (2022). We performed immunostaining for viral antigens only in a limited number of organs. Lung immunostaining was not performed because there was no histology suggestive of viral infection.

This and the fact that the cause of death was unknown even after necropsy were limitations of our study. Necropsy of two cats naturally infected with FeMV detected viral antigens in the urinary tract, respiratory epithelial cells, brain astroglia, and oligodendroglia (Chaiyasak et al. 2022). In this study, we showed that FeMV was frequently detected in cats with acute febrile conditions of unknown etiology. However, we did not determine whether FeMV caused acute infections. Feline calicivirus infection and feline infectious peritonitis are well-known causes of fever in cats. Most of our cases showed acute symptoms suggestive of SFTS and had no upper respiratory tract symptoms; therefore, it is unlikely that they had these infections. Another limitation of this study was that we could not rule out infections with pathogens that cause acute febrile infections in cats. Viral isolation and experimental infection are required to determine FeMV virulence. Several research groups have succeeded in isolating FeMV; however, it was difficult to isolate the virus under common culture conditions (Donato et al. 2021; Woo et al. 2012). Blind passages are required for isolation, during which the virulence of the virus may change. Further epidemiological studies including other FeMV-endemic areas worldwide may reveal the pathogenicity of FeMV in cats. Some studies have reported a high prevalence of FeMV in urinalysis samples (Mohd Isa et al. 2019; Park et al. 2016). In our necropsied case, viral antigens were detected in the renal interstitium and tubular epithelium. Persistent infections can be established in the urinary system, and contact with urine is a presumed main route of transmission. In our study, however, oral swabs and anal samples were available for some cases, and FeMV was also detected in these specimens (Table 1). Recent studies have shown that dogs and wild animals are susceptible to FeMV (Lavorente et al. 2022; Piewbang et al. 2022). It is possible that FeMV is prevalent in cats and other animal species. Respiratory symptoms have also been reported in dogs, which suggests that transmission via droplets and feces may also be considered in acute phase infection.

We designed real-time PCR primers and a probe based on GenBank-registered nucleotide sequences. However, consensus nucleotide sequences suitable for primer design were limited because of the diversity of the virus genome. To improve the accuracy of the PCR test for FeMV, it may be necessary to investigate the nucleotide sequences of viruses found in the target area and prepare a primer set that matches them.

## Data Availability

The datasets generated during and/or analyzed during the current study are available from the corresponding author on reasonable request.
